# Record of Foraminifera test composition throughout the Phanerozoic

**DOI:** 10.1098/rspb.2025.0221

**Published:** 2025-04-09

**Authors:** Katherine Faulkner, Christopher Lowery, Rowan Clare Martindale, Carl Simpson, Andrew Jeffrey Fraass

**Affiliations:** ^1^ Earth and Planetary Sciences, University of Texas at Austin, Austin, TX, USA; ^2^ Department of Earth Sciences, University of Oxford, Oxford, UK; ^3^ Institute for Geophysics, University of Texas at Austin, Austin, TX, USA; ^4^ Department of Geological Sciences, University of Colorado Boulder, Boulder, CO, USA; ^5^ Department of Earth and Ocean Sciences, University of Victoria, Victoria, British Columbia, Canada

**Keywords:** Phanerozoic, Foraminifera, benthic, calcium carbonate, marine ecosystems

## Abstract

Marine calcifiers produce calcareous structures (e.g. shells, skeletons or tests) and are therefore sensitive to ocean chemistry. Nevertheless, the long-term evolutionary consequences of marine carbonate changes are not well understood. This article compares calcareous and non-calcareous responses to ocean chemistry changes throughout the Phanerozoic Eon (541 million years ago to present). To accomplish this, we calculated proportional wall-type diversity, origination rates and extinction rates for 2282 benthic foraminiferal genera. Calcareous origination and extinction rates fluctuated throughout the Palaeozoic Era (541–251.9 million years ago), but during the Mesozoic Era (251.9–66 million years ago), calcareous origination and extinction rates stabilized following the evolution of pelagic calcifiers. Despite variations in Cenozoic Era (66–0 million years ago) foraminifera diversity, calcareous wall types maintained around 77% proportional diversity. Although calcareous wall-type extinction rates decline during the Mesozoic and Cenozoic, Phanerozoic foraminifera wall-type changes during individual events are largely contingent upon contemporaneous conditions rather than overarching trends. Of the Big Five mass extinction events, calcareous wall-type proportions only decreased at the end-Permian (73% to 26% diversity) and end-Triassic (56% to 50% diversity). These results suggest long-term ocean chemistry changes were not the main driver of foraminiferal wall-type diversity through time.

## 1. Introduction

Calcium carbonate (CaCO_3_) is precipitated in the oceans through naturally abundant calcium and dissolved inorganic carbon. Marine organisms, like corals and bivalves, use CaCO_3_ to metabolically produce shells and skeletal structures [1]. Increasing ocean acidification caused by carbon emissions decreases both ocean pH and the carbonate ion saturation state [2,3]. This suggests marine calcifiers may go extinct or suffer physiological effects as anthropogenic carbon emissions increase [4]. Therefore, constraining the response of calcifying and non-calcifying organisms to changes in ocean carbonate chemistry is essential to understanding their sensitivity and resilience.

Specifically, the fossil record throughout the Phanerozoic Eon, from 541 million years ago (Ma) to present, provides numerous examples of short- and long-term changes in oceanic carbonate chemistry [5]. While ocean chemistry has influenced the biotic composition of reef ecosystems [6] and acidification has been linked to several biotic crises [7], the overall impact of acidification on marine calcifiers has proven variable [8,9]. This may, in part, result from evolutionary adaptations to long-term changes in the marine carbonate system. For instance, marine carbonate production was influenced by the rise of pelagic calcifiers in the Mesozoic Era (251.9−66 Ma) [5,10,11] and the Mesozoic Marine Revolution (MMR) evolutionary arms race, which is characterized by the evolution of thick, ornamented shells and infaunal (burrowing) prey [12]. Looking at the ecological trends of carbonate organisms throughout the Phanerozoic may clarify how and why communities respond variably to significant changes in ocean carbonate chemistry.

Foraminifera are an ideal proxy for palaeoecological responses to marine carbonate chemistry since they are globally abundant throughout the fossil record [13]. Their tests (shells) can be constructed from surrounding sediment (agglutinated), biomineralized from CaCO_3_ (calcareous) or constructed from organic material (organic) [14]. Studying changes in foraminifera wall-type diversity during environmental perturbations can provide insights into general responses throughout the marine ecosystem [15–17]. While long-term responses to changing ocean chemistry exist for reef-forming organisms [7] and calcareous plankton [18], less is known about benthic foraminiferal responses. Benthic foraminifera are especially important because their fossil record extends 541 million years [13], compared to planktic foraminifera that originated approx. 175 million years ago [19]. Moreover, while planktic foraminifera are only calcareous, benthic foraminifera have calcareous, agglutinated and organic wall types. Although Phanerozoic shifts in foraminifera diversity are well documented [20,21], few studies have categorized foraminifera into wall types (i.e. agglutinated versus calcareous). Studies that do categorize wall types are only at a family-level resolution [22].

This is the first Phanerozoic-wide study to determine how foraminiferal wall types varied with ocean carbonate chemistry. Laboratory experiments exposing extant benthic foraminifera to ocean acidification parameters found species respond variably, but total diversity decreases [23,24]. Because of this, we expect a loss of calcareous foraminifera diversity during acidification events, and an increase in calcareous test diversity once the CaCO_3_ saturation state stabilizes during the MMR. We also expect overarching changes in calcareous wall types based on shifts between calcite and aragonite seas. Interestingly, we find that this is not the case and that changes in wall-type diversity are largely contingent upon contemporaneous conditions rather than Phanerozoic-long trends.

## 2. Methods

### (a) Data retrieval

Data were retrieved from *Foraminiferal Genera and their Classification* [25], which is still the most current exhaustive list of foraminiferal genera published. While there have been taxonomic revisions since the 1980s [26,27], incorporating piecemeal revisions would introduce phylogenetic and epoch-level bias. Focusing on the opinions and categories of two researchers (i.e. Alfred Loeblich and Helen Tappan) provides consistency between different epochs, which is important for understanding general trends throughout this temporally large dataset. We recorded the order, suborder, superfamily, family, subfamily and genus along with origination interval, extinction interval and wall type. This yielded a total of 3114 taxa classifications. Higher classifications do not impact our work, so we constrained the dataset to genera only. In total, we recorded 2442 genera (160 planktic and 2282 benthic). We use epoch-level resolution since the first and last occurrences in Loeblich & Tappan [25] are inconsistently listed between stage and epoch level.

### (b) Categorizing wall types

The numerous wall-type categories identified in previous literature (e.g. aragonitic, porcelaneous and calcareous-agglutinated) make it challenging to identify meaningful trends and do not appear to be consistently applied. For example, calcareous-agglutinated wall types account for, at most, 1.5% of genera in an epoch. For this reason, we merged reported wall types into one of three groups: calcareous, agglutinated and organic (table 1). We defined a wall type as calcareous if the test precipitated CaCO_3_ from the surrounding environment and agglutinated if the test aggregated materials from the surrounding sediments (even if those agglutinated materials happen to be calcareous). Original wall types are recorded in the dataset presented in the electronic supplementary material, so further division is possible in future research.

**Table 1.** Wall type categorization used in this study as compared to the Loeblich & Tappan [25] descriptions.

wall type described in Loeblich & Tappan	simplified wall type (used in this study)
agglutinated	agglutinated
calcareous and agglutinated	
calcareous, porcelaneous and agglutinated	
porcelaneous and agglutinated	
unknown (*Saudia*, *Sornayina*)	
aragonite	calcareous
calcareous	
porcelaneous	
calcareous and porcelaneous	
organic	organic

Most genera reported as ‘unknown’ wall types are larger benthic foraminifera like fusulinids, which built reef structures and lived on carbonate ramps and platforms [28,29]. Although some debate remains about whether this group’s microgranular calcite walls were precipitated or agglutinated [30,31], most authors agree that fusulinids precipitated their calcareous walls [32], so we classified fusulinid taxa as calcareous. Beyond fusilinids, only three undefined genera remained: *Saudia*, *Sornayina* and *Milammellus. Saudia* and *Sornayina* are classified as agglutinated in the World Register of Marine Species database, while *Milammellus* is classified as opaline [33]. As the only opaline genus in this dataset, *Milammellus* was excluded.

### (c) Statistical analyses

We binned diversity occurrences into the time scale developed by Cohen et al. [34]. Using R Statistical Software [35], we calculated foraminiferal diversity throughout each epoch and counted genera as occurring in a bin if it was in one of the following categories: singleton (evolved and died in the interval), boundary crosser (evolved in an earlier interval and died in a later interval), originator (evolved in the interval then died outside the interval) or extinct (evolved in an earlier interval then died in the interval). We determined the total number of genera, total number of genera with calcareous wall types, total number of genera with agglutinated wall types and total number of genera with organic wall types at any given epoch (electronic supplementary material, table S1).

We also calculated origination ($\hat{p}$) and extinction ($\hat{q}$) rates using equations from Foote [36]. The following equations divide the number of benthic foraminifera crossing the bottom and top of boundaries (Nbt) by the number of benthic foraminifera at either the start (Nt) or the end (Nb) of the interval. This is then standardized by epoch (*t*) and converted to logarithmic scale.


(2.1) $$\hat{p}=\frac{-ln\left(\text{Nbt/Nt}\right)}{\Delta t}$$

(2.2) $$\hat{q}=\frac{-ln\left(\text{Nbt/Nb}\right)}{\Delta t}$$

Null hypothesis bootstrapping for expected origination and extinction rates uses code from Fraass et al. [19]. This code randomly selected genera within our dataset for 1000 samples and determined the expected range of originations and extinctions (within 95% CI) throughout the Phanerozoic. Calcareous groups and agglutinated groups were subsampled for a wall-type specific analysis. All data were plotted at the midpoint of the corresponding epoch.

Given substantial differences in preservation between the earliest and latest parts of the Phanerozoic, caution must be used when comparing, for example, trends in the early Palaeozoic with trends in the Neogene. We calculated the relative proportion of foraminifera wall type through time to account for preservation bias.

We ran a generalized linear model using a square-root link and Gamma variance to track benthic foraminiferal responses to long-term changes. The analysis was between time (million years ago, Ma) and one of the following response variables: calcareous origination rate, calcareous extinction rate, agglutinated origination rate or agglutinated extinction rate. The data were split into the Palaeozoic Era and the Mesozoic + Cenozoic eras to maintain a similar number of datapoints. The origination and extinction rate regressions for each era can be found in the electronic supplementary material (figure S4). We did not include any intervals with a non-positive origination or extinction rate.

## 3. Results

### (a) General trends throughout the Phanerozoic Eon

**Figure 1.**
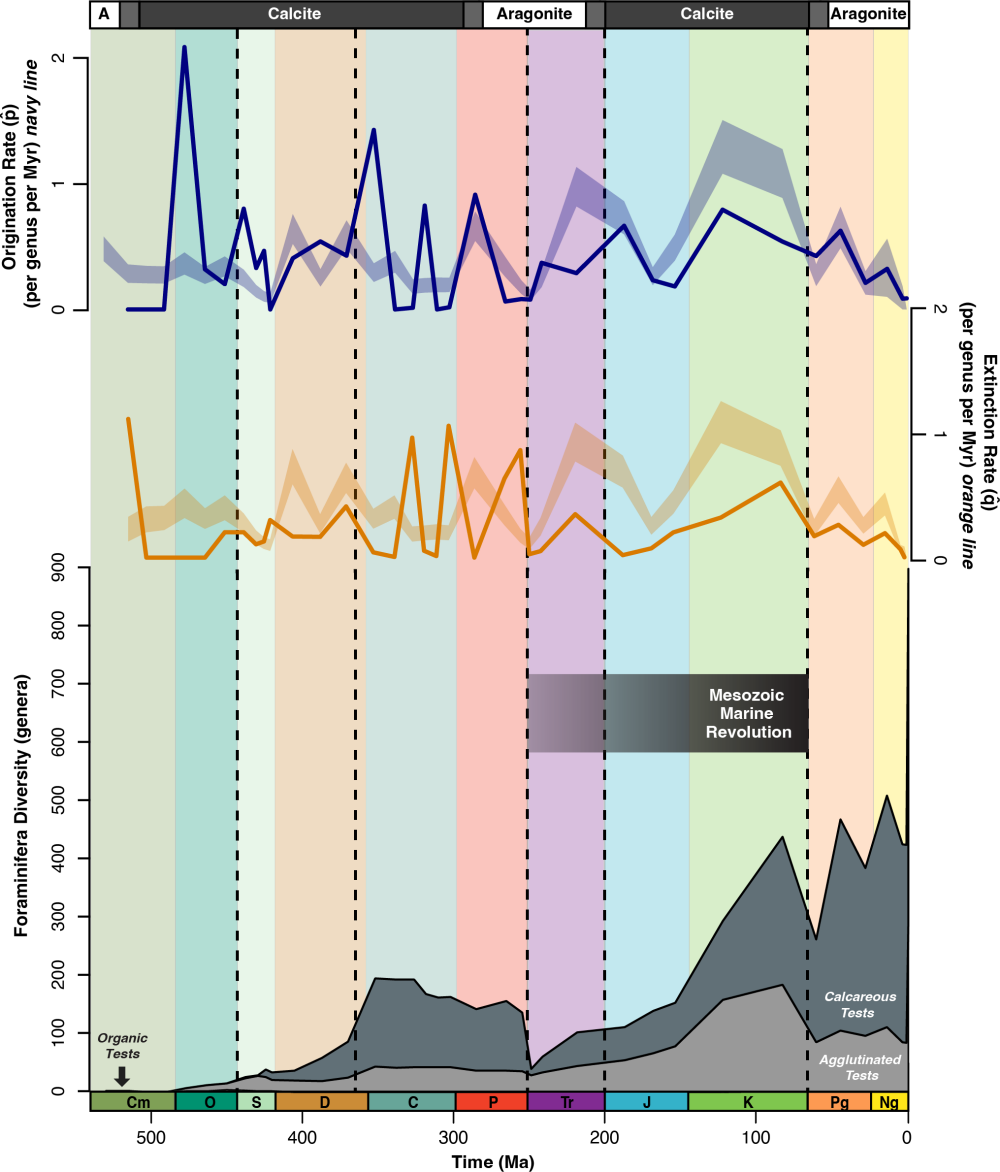
Wall-type diversity, origination rates and extinction rates throughout the Phanerozoic. Foraminifera origination (top, dark blue) and extinction (middle, dark orange) rates in the dataset compared to the expected values within a 95% CI range (blue shading = origination; orange shading = extinction) throughout the Phanerozoic Eon. The bottom plot is generic diversity of Phanerozoic foraminifera [25] separated by wall type (dark grey = calcareous; light grey = agglutinated; off-white (not visible) = organic). Vertical dashed lines indicate the Big Five mass extinction events [37]; the Phanerozoic time scale is at the bottom of the figure; transitions between calcite and aragonite seas are indicated at the top of the figure and the black gradient bar (middle) represents the approximate duration of the Mesozoic Marine Revolution (MMR). *p̂* = origination rate; *q̂* = extinction rate.

**Figure 2.**
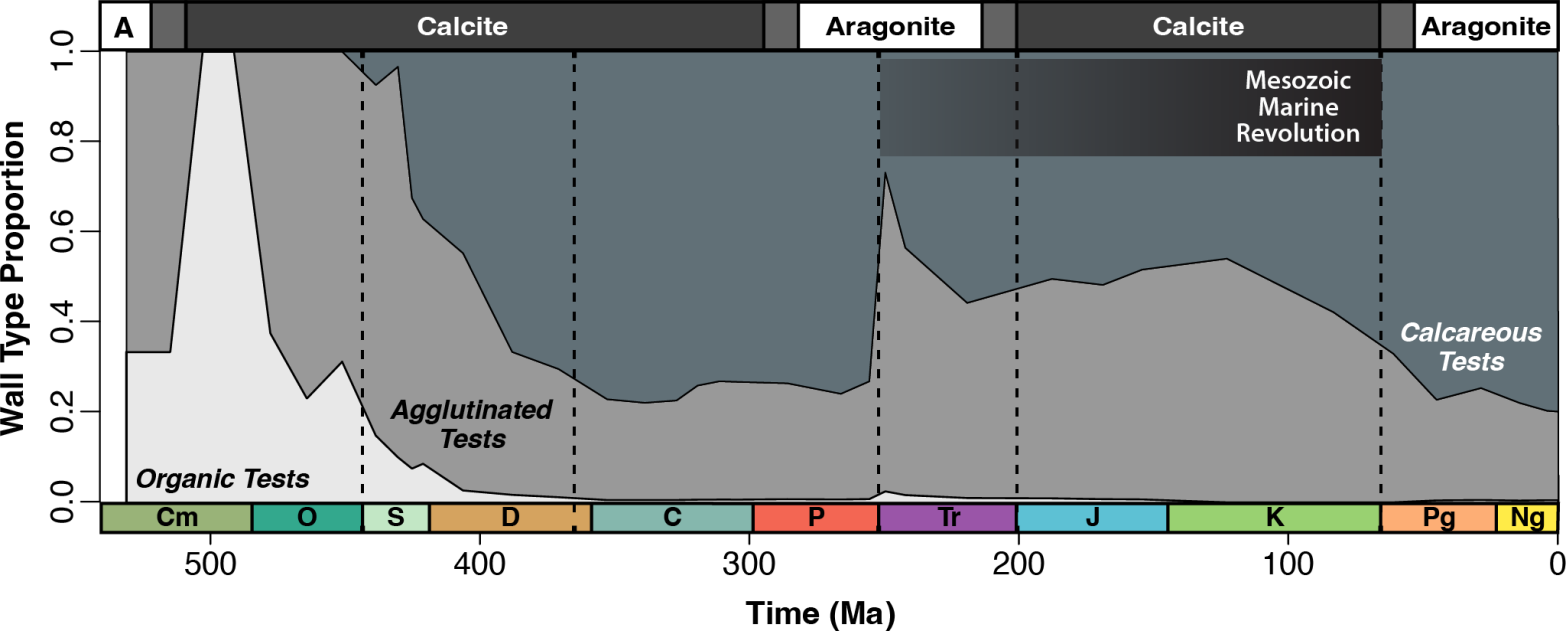
Stacked relative proportion (percentage of genera) of foraminiferal wall types throughout the Phanerozoic Eon. Dark grey = calcareous; light grey = agglutinated; off-white = organic; transitions between calcite and aragonite seas are indicated at the top of the figure. Big Five mass extinction events [37], time scale, transitions between calcite and aragonite seas and Mesozoic Marine Revolution (MMR) all annotated as in figure 1.

Three foraminiferal genera evolved at the beginning of the Cambrian: two agglutinated wall types (*Platysolenites* and *Spirosolenites*) and one organic wall type (*Chitinodendron*). Agglutinated genera rapidly diversified during the Ordovician (485–443 Ma; figures 1 and 2). Calcareous tests first evolved in the early Silurian (444–433 Ma) and diversified to 56 genera by the Late Devonian (383–359 Ma). Calcareous tests remained the highest wall-type proportion from the Late Devonian to the late Permian (between 70% and 77% diversity), after which the end-Permian mass extinction (252 Ma) reduced calcareous test diversity to 11 genera (89% decrease) (figure 1). During the Triassic (252–201 Ma) and Jurassic (201–145 Ma), calcareous tests partially rebounded to 50% of total foraminifera diversity; however, calcareous tests did not fully recover to Palaeozoic diversity levels (>150 genera) until the Late Cretaceous (100–66 Ma). Moreover, calcareous wall types did not fully regain their Palaeozoic proportionality (77% diversity) until the Eocene (56–34 Ma).

### (b) Palaeozoic Era (541−251.9 Ma) mass extinction events

The first two mass extinctions of the Big Five [37] did not greatly impact foraminiferal diversity. Although the end-Ordovician mass extinction (444 Ma) caused 85% of marine life to become extinct [38], agglutinated foraminiferal diversity nearly doubled from 11 to 21 genera (figure 1). Meanwhile, organic wall-type diversity slightly decreased from 5 to 4 genera (figure 1). Across the Devonian (419–359 Ma), foraminiferal diversity increased from 38 genera in the Early Devonian to 88 genera in the Late Devonian (131% increase), and calcareous foraminifera increased from 17 to 62 genera (265% increase) (figure 1). The Late Devonian biotic crisis (approx. 370 Ma) resulted in more extinctions of calcareous tests (37 genera extinctions) compared to agglutinated tests (four genera extinctions), but originations of both groups outpaced extinctions, and total calcareous diversity increased (figures 1 and 2). By the Carboniferous (359–299 Ma), calcareous forms represented around three-quarters of all foraminiferal genera (figure 2).

In contrast to the first two Big Five mass extinctions, the Permian/Triassic mass extinction strongly reduced calcareous foraminifera (figure 2). The eruption of the Siberian Traps is thought to have driven severe ocean acidification and the extinction of 90% of marine life [39–41]. In total, 100 genera went extinct during the Lopingian epoch (259–251 Ma), and calcareous taxa were 23 times more likely to go extinct than other wall types (figures 1 and 2). This was the first time since the Early Devonian (160 Ma earlier) that agglutinated wall types were proportionally greater than calcareous forms (figure 2).

### (c) Mesozoic Era trends (251.9−66 Ma)

After the Permian/Triassic mass extinction, calcareous taxa were at their lowest diversity since the Silurian; however, both calcareous and agglutinated tests increased in diversity throughout the Triassic. Calcareous foraminifera diversified in the Middle Triassic and became the predominant form by the Late Triassic (237–201 Ma; 104 genera) (figures 1 and 2). The Triassic/Jurassic extinction (201 Ma) had little impact on foraminifera wall types (figures 1 and 2). Diversity increased primarily from agglutinated tests (45 to 55 genera), leading to a total net increase of 9 genera, including the evolution of aragonite-only tests in the Early Jurassic.

**Figure 3.**
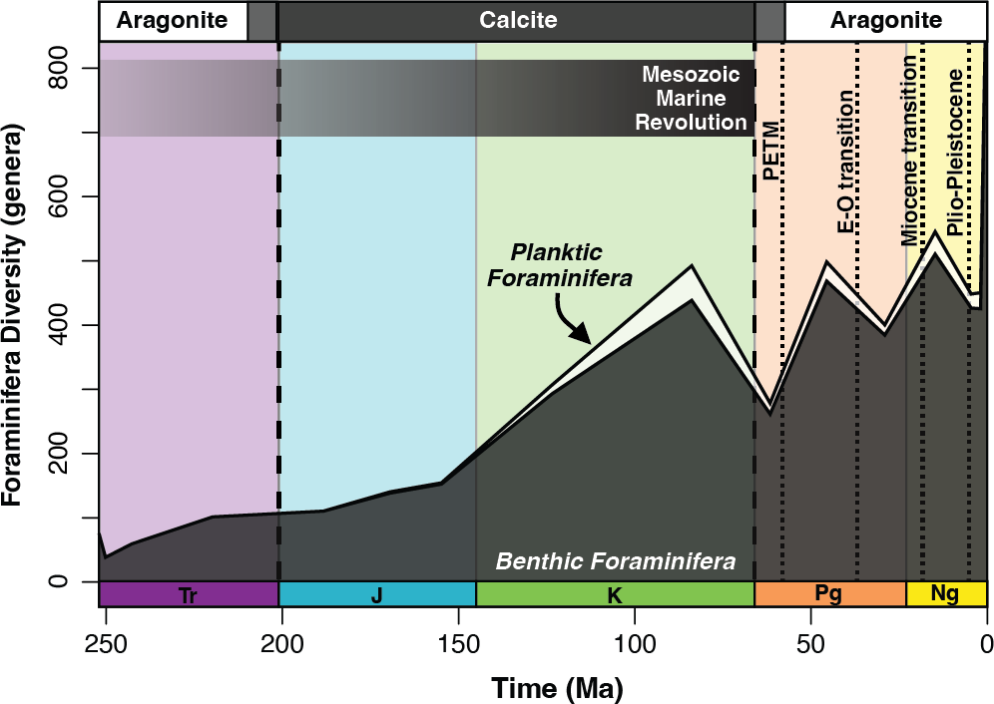
Diversity of foraminifera throughout the Mesozoic and Cenozoic. White = planktic genera; dark grey = benthic genera. Mass extinction events [37], time scale, transitions between calcite and aragonite seas and Mesozoic Marine Revolution (MMR) all annotated as in figure 1; the dotted lines represent Cenozoic warming and cooling events (PETM = Palaeocene−Eocene Thermal Maximum; E-O = Eocene−Oligocene).

Planktic foraminifera first appeared in the Jurassic [19], as did benthic wall types composed of only aragonite. Planktic genera did not noticeably impact total foraminiferal diversity until the Cretaceous (145–66 Ma). Even at their peak diversity in the Late Cretaceous, planktic foraminifera never exceeded 11% of foraminifera genera (figure 3).

**Figure 4.**
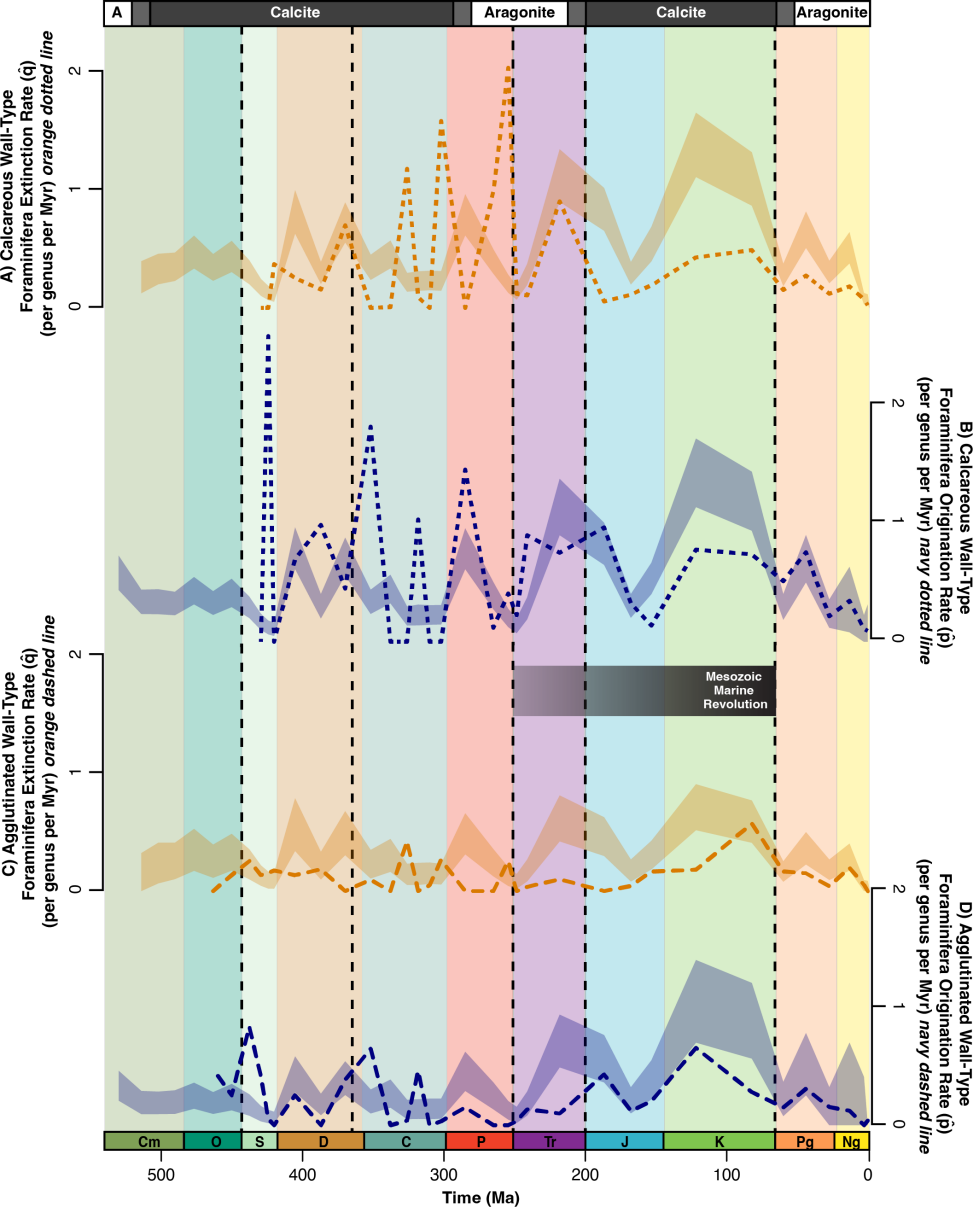
Bootstrap analysis for the origination rates and extinction rates of calcareous (dotted lines) and agglutinated (dashed lines) foraminifera. (A) Expected (light orange shading) versus actual (dark orange dotted line) calcareous extinction rates, (B) expected (light blue shading) versus actual (dark blue dotted line) calcareous origination rates, (C) expected (light orange shading) versus actual (dark orange dashed line) agglutinated extinction rates and (D) expected (light blue shading) versus actual (dark blue dashed line) agglutinated origination rates. Vertical dashed lines indicate the Big Five mass extinction events [37]; the Phanerozoic time scale is at the bottom of the figure; transitions between calcite and aragonite seas are indicated at the top of the figure and the black gradient bar (middle) represents the approximate duration of the Mesozoic Marine Revolution (MMR). *p̂* = origination rate; *q̂* = extinction rate.

During the Cretaceous, both agglutinated and calcareous taxa increased, and their relative proportions were nearly even (figure 2). Several oceanic anoxic events occurred during the Cretaceous [42,43], and agglutinated wall types proportionally increased to a maximum of 54% total diversity in the Early Cretaceous. While the epoch-level resolution of this data makes it difficult to ascribe trends to these short-term oceanic anoxic events, there was a general increase in the diversity of agglutinated tests during this period (figures 1 and 2).

The end-Cretaceous mass extinction (66 Ma) is the most recent of the Big Five mass extinctions and is the only one confidently associated with a bolide impact, the Chicxulub impact [44–46]. In the epoch-level data, there was a 40% net decrease in foraminiferal diversity (441–264 genera), and agglutinated diversity nearly halved from the Late Cretaceous to the Palaeocene (figure 1). While the relative proportion of agglutinated foraminifera decreased from 42% to 33%, calcareous tests proportionally increased from 58% to 67% across the Mesozoic-Cenozoic transition (figure 2).

### (d) Cenozoic Era trends (66–0 Ma)

Throughout the Cenozoic, calcareous tests were the predominant wall type and maintained a low extinction rate (figures 1, 2 and 4A). Total diversity oscillated between each epoch, but there is a general trajectory of diversification (figures 1 and 3). Higher diversity occurred during warmer epochs like the Eocene (471 genera; 56−34 Ma) and Miocene (513 genera; 23−5 Ma); lower diversity levels occurred during colder epochs like the Oligocene (387 genera; 34−23 Ma) and Pliocene−Pleistocene (approx. 430 genera; 5−0.01 Ma) (figure 3). Despite rapid climate fluctuations throughout the latter half of the Cenozoic, foraminifera wall types maintained a constant relative proportion (approx. 80% calcareous and approx. 20% agglutinated) throughout the Eocene−Pleistocene (56 Ma total) (figure 2).

From the Pleistocene to Holocene (2.5−0 Ma), there is a pull of the recent effect [47], reflecting preservation bias, evident as diversity increases from 428 to 869 foraminifera (103% increase), including genera that evolved within the Holocene (442 of 869 genera). When removing singletons from the Holocene, there is a near 0% change in total diversity (428 to 427 genera). The modern oceans contain 246 agglutinated genera (28%), 571 calcareous genera (66%) and 51 organic genera (6%) (figures 1 and 2). Of the 571 calcareous genera, 1 contains both aragonite and calcite and 15 are exclusively aragonite.

### (e) Wall-type origination and extinction rates

During the Palaeozoic, calcareous origination and extinction rates are an order of magnitude greater than agglutinated rates. This difference is especially apparent at the end-Permian mass extinction where calcareous wall types have a considerably higher extinction rate (figure 4A,C). After the end-Permian mass extinction, calcareous wall-type origination and extinction rates decreased by an order of magnitude (figure 4A,B). Moreover, there is almost never a higher-than-expected rate of change for either wall type. The only exception is the origination rate of calcareous wall types in the Early Triassic and Palaeocene (figure 4B). Agglutinated extinction rates and calcareous and agglutinated origination rates reach expected levels by the end of the Cenozoic (figure 4B–D), but calcareous extinction rates consistently stay below the null model (figure 4A).

**Figure 5.**
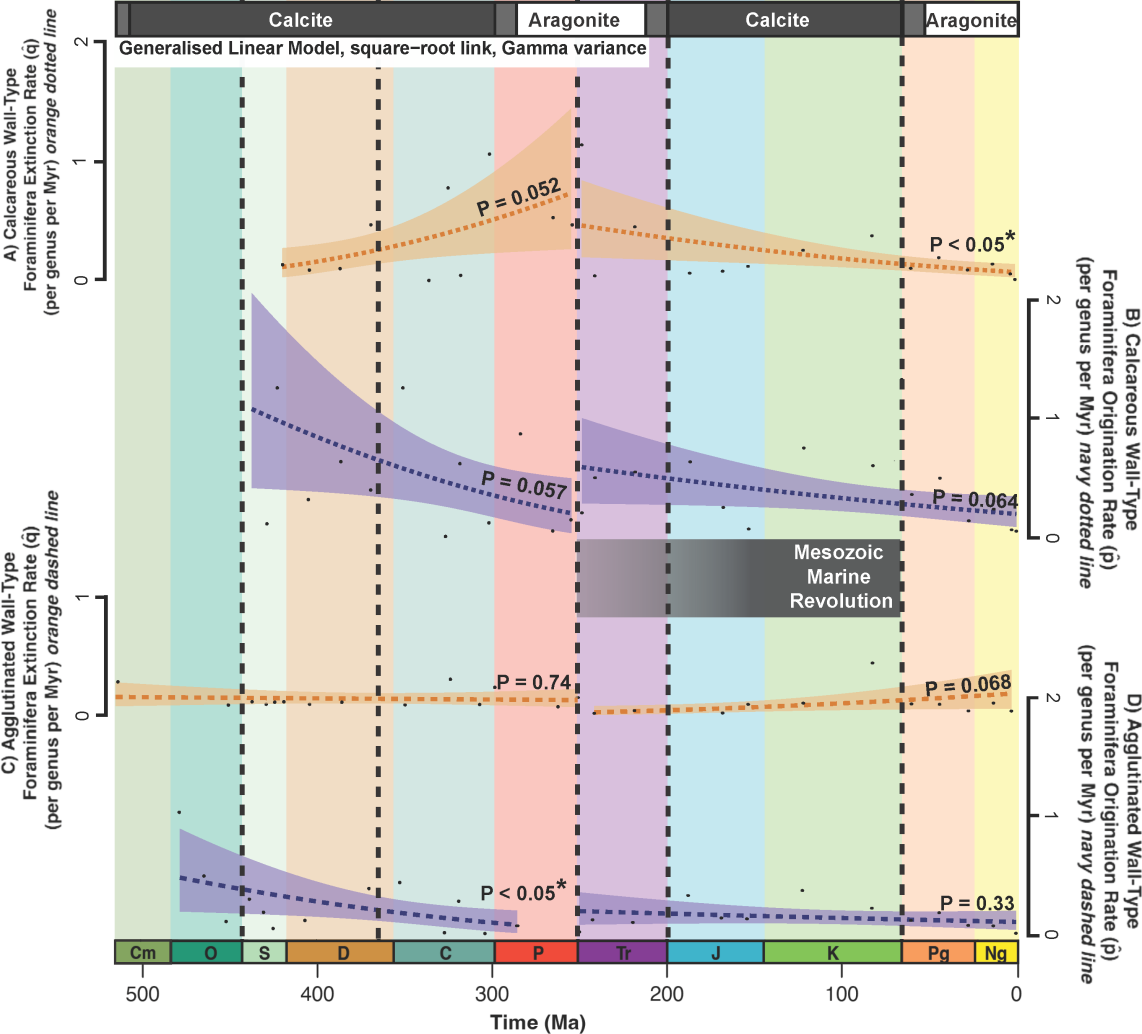
Generalized linear model (square-root link, Gamma variance) for wall-type origination and extinction rates during the Palaeozoic Era and during the Mesozoic + Cenozoic eras. (A) Regression (dark orange dotted line) and variance (light orange shading) of calcareous extinction rates, (B) regression (dark blue dotted line) and variance (light blue shading) of calcareous origination rates, (**C**) regression (dark orange dashed line) and variance (light orange shading) of agglutinated extinction rates and (D) regression (dark blue dashed line) and variance (light blue shading) of agglutinated origination rates; asterisk (*) = statistically significant correlation between interval and wall-type origination/extinction rate. Big Five mass extinction events [37], time scale, transitions between calcite and aragonite seas and Mesozoic Marine Revolution (MMR) all annotated as in figure 1.

We find low correlation between origination/extinction rates and the corresponding time interval in the generalized linear model grouped by the Palaeozoic and Mesozoic/Cenozoic (figure 5). The low *p*-value suggests a significant decrease in agglutinated origination rates throughout the Palaeozoic, but 9 of the 13 points are outside of the generalized linear model standard error (figure 5D). There may be a significant decrease in calcareous rates during the Mesozoic and Cenozoic (figure 5A).

## 4. Discussion

### (a) Palaeozoic Era trends (541−251.9 Ma)

Calcareous wall types were not negatively affected by the Late Devonian mass extinction (figures 1 and 4A,B), and their extinction rates coincide within the extinction rate null model (figure 4A). This differs from corals and coralline sponges, which had a high extinction rate in the Late Devonian [7]. One reason for the disparity could be habitat; many early Palaeozoic foraminifera were infaunal [48], which may have allowed for better adaptation to low-oxygen conditions experienced during the end-Ordovician and Late Devonian mass extinction events [48–50]. Another possible explanation is that the evolution of calcareous wall types coincided with the Silurian greenhouse climate and an increased carbonate saturation state [5,51]. These conditions likely aided the rapid increase in calcareous wall types, which became dominant by the mid-Devonian (figure 2).

A turnover in calcareous wall types at the end of the Devonian (37 extinctions and 30 originations) continued the numerical and proportional increase in calcareous diversity (figures 1 and 2). Increasing carbonate platform areas and decreasing sea level may have aided calcareous wall-type diversification [51]. Abundant carbonate habitats and rising surface carbonate saturation states [5] would have benefited calcareous foraminifera diversification.

Unlike the Late Devonian mass extinction, calcareous wall-type diversity decreased significantly during the end-Permian mass extinction (figures 2 and 4), with the extinction of 93 calcareous genera and 9 agglutinated genera (figures 1 and 2). Calcareous test extinctions coincide with broader selectivity against carbonate skeletons, including corals [6,52–54]. The severity and rapid onset of this extinction likely influenced the decline of CaCO_3_ organisms. Calcareous shells are sensitive to undersaturation of CaCO_3_ [55], and high calcareous extinction rates (figures 1, 2 and 4A) reflect the lack of a carbonate buffer during end-Permian ocean acidification [56]. Recently, the role of acidification at the end-Permian has been questioned due to the confluence of multiple extinction mechanisms, such as rapid warming and expanded oxygen minimum zones [57]; however, neither of these kill mechanisms account for the selection against carbonate foraminifera in our dataset.

Overall, benthic foraminifera responded unpredictably to Palaeozoic perturbations. Agglutinated extinction rates were especially variable with distinct peaks around 520, 320 and 250 Ma (figure 5C). Moreover, while the generalized linear model for agglutinated origination rates has a low *p*‐value, 9 of the 13 data points are outside the model variance (figure 5D), suggesting the model oversimplifies the trend. Therefore, there is no clear evidence of long-term patterns affecting benthic foraminifera. Instead, Palaeozoic benthic foraminifera appear more vulnerable to short-term trends such as environmental changes and CaCO_3_ fluctuations.

### (b) Mesozoic Era trends (251.9−66 Ma)

Although calcareous origination rates were high in the early Mesozoic (figure 4B), their proportional diversity only partially recovered (figure 2). These conflicting results may be due to Early Triassic environmental conditions. Shallow marine anoxic conditions persisted for 5 million years after the Permian/Triassic, which led to a slow recovery of marine communities throughout the Triassic [58]. Reef recovery was particularly slow, with the proliferation of reef ecosystems delayed into the Middle to Late Triassic [59]. This coincided with a slow recovery of calcareous and agglutinated foraminifera in the Late Triassic (figure 2) [27].

Although the end-Permian and end-Triassic kill mechanisms were similar, these two mass extinctions have fundamentally different impacts on benthic foraminifera wall types (figures 2 and 4). Both mass extinction events were caused by pulsed volcanism with strong evidence for ocean acidification [7], but the end-Triassic event resulted in low extinction rates for both calcareous and agglutinated benthic foraminifera (figure 4A,C). The Triassic/Jurassic mass extinction is associated with the Central Atlantic Magmatic Province [60]. The emplacement of these flood basalts substantially increased atmospheric carbon dioxide [61], potentially resulting in ocean acidification [62]. It has been suggested that Triassic/Jurassic acidification hindered calcareous taxa, particularly scleractinian corals, in reef communities [62,63]. The end-Triassic extinction, however, had a net loss of just one genus of calcareous foraminifera (58 to 57 genera) (figure 1).

The difference in severity between the end-Permian and end-Triassic mass extinction events for foraminifera may be caused by the degree and rate of pCO_2_ (atmospheric carbon dioxide) emissions. At the Permian/Triassic mass extinction, pCO_2_ levels increased sixfold [64], whereas pCO_2_ levels increased two- to threefold during Triassic/Jurassic eruption pulses [61]. The unique impact of these two events has also been demonstrated more broadly with palaeocommunities exhibiting limited community change across the Triassic/Jurassic extinction due to increased reef modularity [65,66]. One potential caveat is that there has been a plethora of research focused on the end-Triassic extinction after the publication of Loeblich & Tappan [25], and future work should compile newer data on foraminiferal genera across this interval. Nevertheless, of the Big Five mass extinctions, the proportion of calcareous foraminifera only decreased during the end-Permian and end-Triassic mass extinction events, although only slightly in the latter case (figure 2). Both mass extinctions have evidence of acidification [7], suggesting calcareous wall types may be susceptible to ocean acidification when paired with other kill mechanisms, such as extreme temperatures and deoxygenation.

Our data indicate both calcareous and agglutinated foraminiferal diversity increased during the MMR. This coincides with several reef booms [66] and low extinction rates for corals and coralline sponges from the mid-Jurassic to Early Cretaceous [7]. By the Late Cretaceous, calcareous wall-type extinctions were lower than expected (figure 4A), and more genera had calcareous tests than any other type (figures 1 and 2). These findings may be explained by the transition in the ocean carbonate system during the MMR. Palaeozoic ocean carbonate chemistry was controlled by benthic carbonate producers [5], which may account for the sharp fluctuations in calcareous wall-type originations and extinctions before the Mesozoic (figure 1). During the Mesozoic, the evolution of pelagic calcifiers, like nannoplankton and planktic foraminifera, stabilized the ocean carbonate cycle [5,67], and reef carbonate accretion increased [66]. The widespread deposition of pelagic carbonates [5,11] created a deeper and more distributed carbonate flux in the world’s oceans. Consequently, calcareous foraminifera, and other marine calcifiers, were more resilient to rapid environmental perturbations (figures 1 and 2) [7]. Although some genera still went extinct during intervals like the Toarcian Oceanic Anoxic Event [68], overall calcareous test diversity increased by 28% from the Early to Middle Jurassic (201–163 Ma), and the number of coral reef sites increased by the Late Jurassic [6]. This signals more resilience to changing ocean conditions when compared with previous events (figures 1 and 4).

The Cretaceous/Palaeogene (K/Pg) is the only Big Five mass extinction aside from the end-Permian to cause a significant decrease in total foraminifera diversity (approx. 40% of foraminiferal genera; figures 1 and 3). While extinction during this interval is often associated with planktic foraminifera [18], benthic foraminifera also lost diversity, albeit less severely (figures 3 and 4). Specifically, larger benthic foraminifera experienced high extinction rates [69,70], but smaller benthic foraminifera were generally resilient across the K/Pg boundary, both in the deep sea and on continental margins [16,71].

Similar to the end-Permian and end-Triassic mass extinction events, the K/Pg mass extinction shows evidence of ocean acidification [72]; however, unlike the end-Permian and end-Triassic mass extinctions, the majority of surviving taxa after the K/Pg were calcareous wall types (figure 2). This coincides with a low extinction rate for corals and coralline sponges [7] and an increase in the number of coral reef sites [6]. Given the resolution of our data, we cannot determine whether this decrease in agglutinated genera occurred at the boundary or across the Late Cretaceous. Agglutinated wall-type diversity probably did not decrease at the boundary since there are no documented extinctions of smaller benthic foraminifera during this time [16,71]. Rather, we suspect that the decline in the proportion of agglutinated genera is part of a long-term trend that continued until the Eocene.

### (c) Cenozoic Era trends (66–0 Ma)

Both long- and short-term trends affected benthic foraminifera throughout the Cenozoic. During this era, origination rates stabilized (figure 4B,D), and calcareous extinction rates decreased (figures 4A and 5A). This corresponds with low coral and coralline sponge extinction rates in the Cenozoic [7]. These patterns may reflect an established carbonate saturation state after the MMR [5], which would allow for the stable ratio of calcareous and agglutinated benthic foraminifera during the Cenozoic (figure 2).

Despite increased carbonate chemistry stability following the MMR, the generalized linear model simplifies the cyclical origination/extinction rates, which vary at epoch level (figures 1 and 4). These fluctuations coincide with warming and cooling events throughout the Cenozoic (figure 3) [18,73]. While stage-level climate shifts are below the resolution of our work, they have a well-documented influence on foraminiferal diversity [16,19,74]. Despite absolute wall-type diversity fluctuating between epochs (figure 1), the proportional diversity of calcareous wall types remained stable (between 74% and 80%) from the Eocene to the Pleistocene (56 Ma−11.7 ka; figure 2). The stability in proportional diversity despite fluctuations in the number of foraminiferal genera suggests a turnover in calcareous wall types throughout the Cenozoic.

The stability of wall-type proportions despite diversity fluctuations may be explained by the abundance of pelagic calcifiers, which would have helped buffer the oceans against rapid alkalinity changes [5,67,72]. Although reef-forming organisms are particularly susceptible to modern ocean acidification, pelagic calcifiers like planktic foraminifera, calcareous nannoplankton and pteropods seem more resilient than other taxa. This is because factors like growth rate and respiration appear unaffected by changes in pH on a scale the oceans are likely to experience [9]. This consistent presence of pelagic calcifiers would allow benthic calcareous wall types to quickly recover during perturbations in ocean chemistry (figures 1 and 2).

### (d) The effects of Mg/Ca ratios on foraminifera throughout the Phanerozoic

Ocean Mg/Ca ratios are important parameters in ocean carbonate chemistry [75], as increased Mg^2+^ ions inhibit calcite nucleation and thus favour aragonite, even though it is less stable than calcite [76]. These long-term shifts are referred to as calcite/aragonite seas (low or high Mg/Ca ratios, respectively) [75]. Our data show no clear relationship between either foraminiferal diversity or dominant wall type and the intervals of calcite/aragonite seas across the Phanerozoic (figures 1 and 2). Although Mg/Ca levels may be important in organism mineralogy during originations [76,77], the shifts between calcite and aragonite seas do not control the dominant test mineralogy or diversity in foraminifera. Although there are conflicting results on the biological effects of aragonite/calcite seas, it is agreed that other factors—such as reef crises, mass extinctions and biological constraints—are more impactful to long-term trends in coral and foraminiferal diversity [6,63,78,79].

### (e) Potential caveats

The early history of benthic foraminifera is difficult to decipher since the time interval is understudied. There is low organic test diversity and a disappearance and resurgence of agglutinated wall types during the Miaolingian and Furongian series of the Cambrian (509–485 Ma). Undersampling is a common issue in the Palaeozoic [80], and targeted research is needed to provide a more comprehensive review of benthic foraminifera diversity in the Cambrian and Ordovician. Moreover, most of the fossil record contains only one organic genus (*Archaeochitinia*) from the Early Devonian to the Late Jurassic, but the Holocene has 51 organic-walled genera, indicating a strong preservation bias.

Additionally, the oldest extant oceanic crust, with some exceptions, is Jurassic in age [81]; prior to this period, the lack of samples from the largest environment on Earth means that the fossil record of benthic foraminifera is especially limited. Abyssal-assemblage preservation in oceanic crust may account for part of the increasing total generic diversity in the later Mesozoic. This preservation bias, along with changes in the carbonate compensation depth, mean that pre-Jurassic events are, at best, incomplete analogues for current and future marine calcifier responses to climatic and oceanographic changes.

Moreover, this dataset is analysed at the generic level, and taxonomic diversity is not equal to abundance. Although species-level data similar to Fenton et al. [82] would be ideal for this study, such occurrence data do not yet exist for benthic foraminifera throughout the Phanerozoic. It is also important to note our dataset is at epoch-level resolution. With mass extinction events, it is possible the recorded extinctions occurred before the boundary and not during the extinction event itself. Although stage-level data would provide a finer resolution, Loeblich & Tappan [25] inconsistently lists genera at either the stage or epoch level. We chose to bin by epochs for consistency, and future work should highlight events at a stage-level scale to amplify more subtle benthic foraminiferal responses during rapid changes like ocean anoxic events.

## 5. Conclusions

By partitioning foraminifera genera into calcareous and agglutinated wall types, we find unique responses to ocean chemistry throughout the Phanerozoic. Palaeozoic calcareous origination and extinction rates were an order of magnitude higher than agglutinated origination and extinction rates. Calcareous wall types rapidly diversified after evolving in the Silurian and became 70% of global wall-type diversity after the Late Devonian mass extinction. This proportional dominance was maintained throughout the Carboniferous and Permian (between 73% and 78% diversity). Of the Big Five mass extinctions, only two events decreased calcareous proportional diversity: the end-Permian (73% to 27% global diversity) and end-Triassic (55% to 50%). In the Mesozoic, calcareous origination and extinction rates were similar magnitudes as agglutinated origination and extinction rates. Although calcareous wall types reached a local minimum in the Jurassic (45% global wall-type diversity), they gradually rebounded from the Cretaceous to the Eocene (57% to 67% global diversity). This calcareous wall-type resilience is likely due to a more buffered ocean, caused by the diversification of Mesozoic pelagic calcifiers. Despite foraminiferal diversity fluctuating throughout the Cenozoic, proportional abundance of calcareous wall types remained stable from the Eocene to the Pleistocene (75% to 80% global diversity). These results correspond with a decrease in calcareous extinction rates throughout the Mesozoic and Cenozoic. Overall, foraminifera responded to short-term ocean chemistry changes throughout the Phanerozoic, but the establishment of the modern carbonate system likely provided more environmental stability for calcareous wall types to flourish and become dominant.

## Data Availability

All data and code can be found within the electronic supplementary material [83].
